# Comparison of Simulated and True Keratometry Measurements Using Swept-Source Optical Coherence Tomography and Dual Scheimpflug–Placido Imaging

**DOI:** 10.1155/2021/5860846

**Published:** 2021-09-16

**Authors:** Elizabeth A. Urias, Efstathia Polychronopoulou, Rahul T. Pandit

**Affiliations:** ^1^Blanton Eye Institute, Houston Methodist Hospital, Houston, TX, USA; ^2^University of Texas Medical Branch, Galveston, TX, USA; ^3^Houston Methodist Eye Associates, Houston, TX, USA

## Abstract

**Purpose:**

To compare simulated and total keratometry and corneal astigmatism values between the IOLMaster 700 and Galilei G4 devices.

**Methods:**

A retrospective chart review was conducted for all patients undergoing phacoemulsification by a single surgeon (RTP) from March through September 2020 and who underwent imaging with both the IOLMaster 700 and Galilei G4. Exclusion criteria were prior corneal surgery, keratectatic diseases and inability to obtain a reliable image during image acquisition. Mean, flat, and steep keratometry values as well as astigmatism magnitude were compared.

**Results:**

A total of 200 eyes of 100 patients were included. Intraclass correlation coefficients (ICC) were moderate or high for all variables. Mean difference ± SD in SimK and TrueK between devices (G4-IOLM) was 0.05 ± 0.318 diopters and −1.1156 ± 0.438 diopters, respectively (*p* < 0.05 for both). The IOLM measured steeper TrueK value than the G4. For SimK, there was a statistically significant difference between devices only for mean keratometry (K), whereas for TrueK, there were significant differences in flat K, steep K, and mean K. Astigmatism analysis revealed a difference in mean (±SD) SimK of 0.07 (±0.57) D at 94 degrees and in mean TrueK of 0.04 (±0.85) D at 108 degrees.

**Conclusion:**

Though there is overall good correlation between the IOLMaster 700 and Galilei G4 in SimK and astigmatism measurements, there is a significant difference in TrueK measurements, with the IOLM measuring steeper values by about 1.0 diopter as compared to the G4.

## 1. Introduction

The ability to measure keratometry with both accuracy and reproducibility is critical to properly screen and provide patients with excellent postoperative refractive outcomes. Historically, keratometry has been limited to only direct measurement of the anterior corneal surface. Conventional, or simulated, keratometry (SimK) theoretically estimates corneal power by using the anterior corneal curvature and the standard refractive index. Newer technologies have allowed us to directly measure the anterior and posterior surface of the cornea to obtain the true corneal power (TrueK).

The IOL Master 700 (IOLM; Carl Zeiss, Jena, Germany) utilizes 18 telecentric spots to calculate anterior corneal curvature and therefore conventional keratometry (IOLM-K), as well as swept-source OCT (SSOCT) biometry which uses a rapid-cycle and tunable wavelength laser light source to also measure corneal pachymetry and posterior corneal curvature in six meridians [1]. Measured curvatures are converted to powers (D) utilizing the refractive indices of the cornea (1.376) and the aqueous (1.336), incorporating pachymetry data to calculate true keratometry (IOLM-TK). IOLMaster 700 has shown high reproducibility for ocular biometry in healthy subjects [2].

In contrast, the Galilei G4 Dual Scheimpflug Analyzer (G4; Ziemer Ophthalmic Systems AG, Port, Switzerland) combines dual Scheimpflug tomography with Placido disk topography to obtain both simulated (G-SimK) and total corneal power TCP_IOL_ (G-TCP). The simultaneously recorded dual Scheimpflug images produce reliable pachymetry and posterior curvature data. However, the Placido ring images provide highly accurate central anterior corneal curvature data. The Galilei G4 aligns all data to the visual axis using the first Purkinge image, which ensures consecutive measurements [3]. High intraobserver repeatability has been demonstrated in the dual Scheimpflug analyzer for simulated total and posterior corneal power measurements and moderate repeatability for posterior corneal astigmatism [4].

The purpose of this study was to compare simulated and total keratometry and corneal astigmatism values between the IOLMaster 700 and Galilei G4 devices.

## 2. Materials and Methods

A retrospective chart review was conducted for all patients undergoing phacoemulsification by a single surgeon (RTP) from March through September 2020. Patients who underwent imaging with both the IOLMaster 700 and Galilei G4 were included. Exclusion criteria were prior corneal surgery, keratectatic diseases, and inability to obtain a reliable image during image acquisition. Institutional Review Board approval was obtained from the Houston Methodist Research Institute, Houston, TX, and this study adhered to the principles of the Declaration of Helsinki. Mean, flat, and steep SimK as well as TrueK (TK from the IOLMaster 700 and TCP_IOL_ from the Galilei G4) were recorded from both devices. Primary endpoints were mean differences in SimK and TrueK. Secondary endpoints were mean differences in both simulated and true corneal flat keratometry, steep keratometry, corneal astigmatism magnitude, and corneal astigmatism as calculated by conversion to cross cylinder. [5].

### 2.1. Statistical Methods

Statistical analysis was performed using SAS 9.4 (Cary, NC, US). Intraclass correlation coefficient (ICC) was used to measure the strength of the association between the two instruments (high >0.90 or moderate 0.75 to 0.90) [6]. *p* values were calculated using paired two-tailed *t*-tests and the nonparametric Wilcoxon test (for those pairs that violated normality) to compare keratometry measurements between the two instruments. A probability of less than 5% (*p* < 0.05) was considered statistically significant. Agreement between the devices was evaluated using Bland–Altman analysis [7]. The 95% limits of agreement (LoA) were calculated using the mean difference ±1.96 standard deviation (SD). Scatter plots were created to evaluate the relationship between corneal dioptric power and difference in keratometry measurements between instruments. Double-angle plots were created to compare astigmatism measurements between devices [8].

Sample size calculation was performed using PASS 2021 software. We calculated that a sample size of 189 achieves 80% power to detect a mean of paired differences of 0.1 D in mean keratometry with an estimated standard deviation of paired differences of 0.4876 and with a significance level (alpha) of 0.05 using a two-sided paired *t*-test.

## 3. Results

A total of 214 eyes of 107 patients were identified. Fourteen eyes (7 patients) were excluded due to prior corneal surgery and/or keratectatic disease. A total of 200 eyes (100 patients) were included in final data analysis. Males comprised 49 patients, and eyes were equally divided between right and left eyes. The mean patient age was 66 years (SD ± 8.9; range 32–85). The mean ± SD, maximum, and minimum keratometry values for all variables measured on the IOLM and G4 are listed in Table 1, along with differences between devices and statistical significance.

The mean ± standard deviation (SD) SimK on the IOLM and G4 were 43.659 ± 1.547 diopters (range 38.94 to 46.730) and 43.710 ± 1.611 diopters (range 38.97 to 46.950; *p* < 0.05), respectively. The mean ± SD TrueK on the IOLM and G4 were 43.740 ± 1.537 diopters (range 39.180 to 46.730) and 42.624 ± 1.606 diopters (range 38.210 to 45.980; *p* < 0.05), respectively (Table 1).

**Figure 1:**
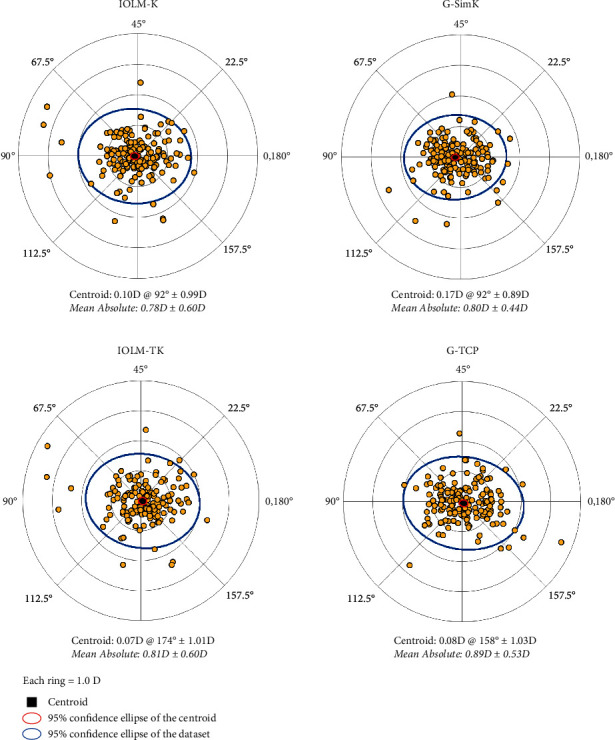
Double-angle plots of astigmatism. IOLM-K = IOLMaster Sim K G-SimK = Galilei G4 simulated keratometry. IOLM-TK = IOLMaster TruK. G-TCP = Galilei total corneal power.

For SimK, there was a statistically significant difference between devices only for mean keratometry (K), whereas for TrueK, there were significant differences in flat K, steep K, and mean K (Table 1). Astigmatism analysis revealed a difference in mean (±SD) SimK of 0.07 (±0.57) D at 94 degrees and in mean TrueK of 0.04 (±0.85) D at 121 degrees. Double-angle plots of SimK and TrueK for both devices are depicted in Figure 1 and show the close agreement in mean astigmatism centroids and 95% confidence ellipses.

Intraclass correlation coefficients (ICC) for all values comparing IOLM and G4 are listed in Table 1. ICC for mean SimK and TrueK measurements between devices was 0.99 and 0.852, respectively. The remaining values also were all moderate or high.

**Figure 2:**
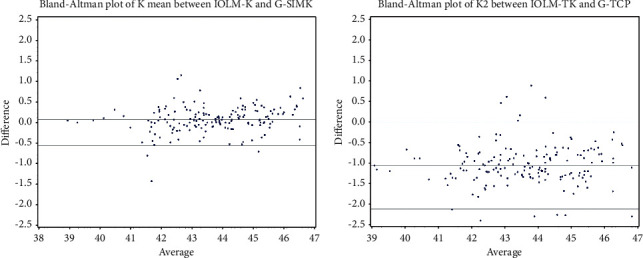
Bland–Altman plots depicting interdevice agreement in primary endpoints mean SimK (a) and mean True K (b). Horizontal lines depict mean ± 1.96 standard deviation (95% LOA) values. *X*-axis = mean corneal power (D). *Y*-axis = difference in mean value (Galilei-IOLMaster).

**Figure 3:**
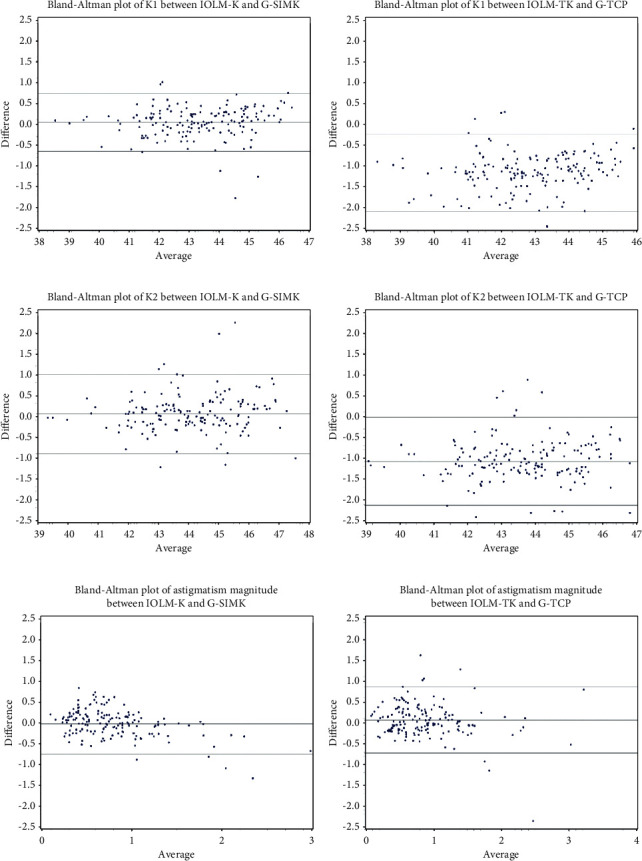
Bland–Altman plots depicting interdevice agreement in secondary endpoints: (a) flat SimK, (b) flat TrueK, (c) steep SimK, (d) steep TrueK, (e) SimK magnitude, and (f) TrueK magnitude. Horizontal lines depict mean ± 1.96 standard deviation (95% LOA) values. *X*-axis = diopters mean corneal power (a–d) and corneal astigmatism (e, f). *Y*-axis = difference in mean value (Galilei-IOLMaster).

Mean difference ± SD in SimK and TrueK between devices (G4 - IOLM) was 0.05 ± 0.318 diopters and −1.1156 ± 0.438 diopters, respectively (both *p* < 0.05; Table 1). The IOLM measured steeper TrueK value than the G4. Bland–Altman plots depicting differences between devices for primary and secondary endpoints are displayed in Figures 2 and 3, respectively.

## 4. Discussion

Our data suggest that there is good overall correlation between the IOLM and G4 devices. Specifically, there was good comparability for all SimK variables as well as corneal astigmatism. We found, however, a systematic and significant difference in TrueK measurements between devices, with the IOLM measuring consistently steeper values than the G4. Though our results demonstrated a small but statistically significant difference between devices in mean SimK, the difference in mean keratometry of 0.05D is clinically irrelevant. There was no statistically significant difference in flat or steep SimK.

Several prior studies have compared correlation and agreement of SimK measurements between the devices we investigated and other newer or older technologies, including IOLMaster 500, Pentacam XL, Verion imaging guiding system, and Sirius Scheimpflug–Placido topographer [1, 9–14]. Similar to our study, a recent prior study evaluated the repeatability and comparability of the IOLMaster 700 with the Galilei G4 [15]. The study included 82 eyes from 48 patients and was powered to detect a difference of 0.2 D in keratometry. In their study, the two devices produced highly correlated and interchangeable SimK values. On the contrary, posterior K values (mean, flat, and steep) were moderately correlated and not interchangeable between devices. Their results demonstrated that the IOLMaster 700 consistently reported steeper TrueK values than the G4, particularly due to measuring flatter posterior corneal curvatures than the Galilei G4.

Similar to this prior study, our study demonstrated a statistically significant and clinically relevant difference of 1.116 diopters in TrueK measurements, with IOLM consistently measuring steeper values than G4. This finding was associated with a statistically significant difference between devices in both flat and steep TrueK values of a similar magnitude. Such a difference in keratometry can result in a difference in choice of intraocular lens by approximately 1.0 diopter. As such, we conclude that TrueK values between the IOLMaster 700 and Galilei G4 are not interchangeable.

We noted, however, that the astigmatism measurements between devices are not significantly different, with a small mean difference and overlapping astigmatic centroids for both SimK and TrueK values. This suggests that the devices may be more similar in measuring magnitude and direction of astigmatism when it comes to TrueK, rather than in total keratometric value.

Of note, in evaluating true keratometry values in the Galilei G4, there are 3 options for total corneal power. The value used in this study, namely, TCP_IOL_, is the current default value for the G4 device. TCP1 was the original value incorporated in the Galilei G1 and carried forward in subsequent device iterations. TCP2 was introduced to try to better estimate true corneal power, and finally, this too was replaced by TCP_IOL_, though all options remain on current devices to allow users the ability to customize individual preferences. TCP1 is based on the corneal index of refraction 1.376, and the reference plane is the anterior corneal surface. TCP2 is based on the aqueous index of refraction 1.336 with the same reference plane of the anterior corneal surface. TCP2 typically results in a value approximately 1.2 D less than TCP1. TCP_IOL_, like TCP2, is also based on the aqueous index of refraction. However, the reference plane is the posterior corneal surface. TCP_IOL_ typically results in values halfway between TCP1 and TCP2 [16]. Though our study did not specifically compare the older TCP1 and TCP2 values, the large mean difference in TruK values between devices suggests that there may be lack of comparability with any TCP value; this in fact was demonstrated in a recent study [15].

Limitations of our study include its retrospective design as well as the fact that it was powered to detect a 0.1 D difference between measurements at a power of 80%. A difference of 0.1 D is of academic interest when comparing devices since such a small difference is clinically insignificant and would not result in a difference in IOL selection for a given patient. As such, this study may be overpowered to detect a clinically significant difference, as exemplified by our statistically significant mean difference of 0.05 D seen in SimK that was deemed clinically insignificant.

## 5. Conclusion

In summary, our data show that though there is overall good correlation between the IOLMaster 700 and Galilei G4 in SimK and astigmatism measurements, there is a significant difference in mean TrueK measurements, with the IOLM measuring steeper values by about 1.0 diopter as compared to the G4. This can result in a difference in choice of IOL by approximately 1.0 diopter. Astigmatism measurements were similar between devices for both SimK and TrueK. Clinicians should refrain from using mean TrueK values interchangeably between these devices. Further studies are needed to determine device performance in corneas with pathology such as keratectatic diseases.

## Figures and Tables

**Table 1:** Mean differences between devices.

Device and Variable	Mean	SD	Min	Max	Mean diff	SD diff	*p* value^*∗*^	ICC
*Primary endpoints*								
Mean IOLM-K	43.659	1.547	38.940	46.730	0.050	0.318	**0.0264**	0.99
Mean G-SimK	43.710	1.611	38.970	46.950				
Mean IOLM-TK	43.740	1.537	39.180	46.730	−1.116	0.438	**<0.0001**	0.852
Mean G-TCP	42.624	1.606	38.210	45.980				

*Secondary endpoints*								
Flat IOLM-K	43.271	1.545	38.520	46.220	0.038	0.353	0.1278	0.987
Flat G-SimK	43.309	1.580	38.600	46.660				
Steep IOLM-K	44.055	1.603	39.370	48.060	0.050	0.485	0.1474	0.977
Steep G-SimK	44.105	1.659	39.330	47.330				
Astigmatism IOLM-K	0.813	0.574	0.000	3.440	−0.032	0.368	0.5117^†^	0.842
Astigmatism G-SimK	0.781	0.409	0.070	2.640				
Flat IOLM-TK	43.337	1.543	38.760	46.190	−1.160	0.471	**<0.0001**	0.84
Flat G-TCP	42.177	1.615	37.860	45.830				
Steep IOLM-TK	44.145	1.595	39.620	47.960	−1.080	0.538	**<0.0001**	0.857
Steep G-TCP	43.065	1.638	38.550	46.240				
Astigmatism IOLM-TK	0.826	0.583	0.000	3.650	0.062	0.405	0.0624^†^	0.846
Astigmatism G-TCP	0.888	0.532	0.020	3.610				

^*∗*^*p* value from paired *t*-test (if normal distribution) or nonparametric test (nonnormal); bold values indicate statistical significance. ^†^indicates *p* value from nonparametric test. IOLM-K = IOLMaster SimK; IOLM-TK = IOLMaster 700 TK; G-SimK = Galilei G4 SimK; G-TCP = Galilei G4 TCPIOL; SD = standard deviation; mean diff = mean difference G4 value minus IOLM value; SD diff = standard deviation of the mean diff; ICC = intraclass correlation coefficient.

## Data Availability

All research data are available upon request.
